# Protective Effect of Black Rice Cyanidin-3-Glucoside on Testicular Damage in STZ-Induced Type 1 Diabetic Rats

**DOI:** 10.3390/foods13050727

**Published:** 2024-02-27

**Authors:** Hongxing Zheng, Yingjun Hu, Jia Zhou, Baolong Zhou, Shanshan Qi

**Affiliations:** 1 School of Biological Science and Engineering, Shaanxi University of Technology, Hanzhong 723000, China; zhenghongxing100@126.com (H.Z.);; 2 State Key Laboratory of Qinba Biological Resources and Ecological Environment, Hanzhong 723000, China; 3 Qinba Mountain Area Collaborative Innovation Center of Bioresources Comprehensive Development, Hanzhong 723000, China; 4 Shaanxi Province Key Laboratory of Bio-Resources, Hanzhong 723000, China; 5 Shaanxi Black Organic Food Engineering Technology Research Center, Hanzhong 723000, China; 6 Shaanxi Guzhongcun Ecological Agriculture Company, Hanzhong 723000, China

**Keywords:** cyanidin-3-glucoside, diabetes, testicular damage, sperm quality, TGF-β1/Smad pathway

## Abstract

Diabetic testicular damage is quite a common and significant complication in diabetic men, which could result in infertility. The natural fertility rate of type 1 diabetes men is only 50% because of testicular damage. This research first aimed to explore the intervention effect of C3G on testicular tissue damage induced by diabetes. Here, a streptozotocin-induced type 1 diabetic rat model was established, and then C3G was administered. After 8 weeks of C3G supplementation, the symptoms of diabetes (e.g., high blood glucose, lower body weight, polydipsia, polyphagia) were relieved, and at the same time that sperm motility and viability increased, sperm abnormality decreased in C3G-treated diabetic rats. Furthermore, the pathological structure of testis was restored; the fibrosis of the testicular interstitial tissue was inhibited; and the LH, FSH, and testosterone levels were all increased in the C3G-treated groups. Testicular oxidative stress was relieved; serum and testicular inflammatory cytokines levels were significantly decreased in C3G-treated groups; levels of Bax, Caspase-3, TGF-β1 and Smad2/3 protein in testis decreased; and the level of Bcl-2 was up-regulated in the C3G-treated groups. A possible mechanism might be that C3G improved antioxidant capacity, relieved oxidative stress, increased anti-inflammatory cytokine expression, and inhibited the apoptosis of spermatogenic cells and testicular fibrosis, thus promoting the production of testosterone and repair of testicular function. In conclusion, this study is the first to reveal that testicular damage could be mitigated by C3G in type 1 diabetic rats. Our results provide a theoretical basis for the application of C3G in male reproductive injury caused by diabetes.

## 1. Introduction

Diabetes is a metabolic syndrome with increased blood glucose [1,2]. The International Diabetes Federation (IDF) reported that the number of diabetes cases will rise to 592 million by 2035 worldwide [3]. Diabetic testicular damage is the most common complication in diabetic men [4,5]. Many men with diabetes are severely affected, both physically and psychologically, due to the reproductive damage caused by diabetes [6,7]. It was reported that the natural fertility rate of type 1 diabetes men is only 50% [8]. Diabetic testicular injury is characterized by testicular atrophy, decreased spermatogonium, and disturbance of sex hormone [9,10,11].

Oxidative stress and inflammatory responses are closely associated with diabetic testicular injury [12,13]. Excessive advanced glycation end products can reduce the supply of glycogen and decrease the spermatogenic ability of testis. High-glucose environments in diabetes, as well as excessive ROS, lead to testicular tissue damage and germ cell apoptosis, as well as reproductive hormone disorder [14,15]. Moreover, up-regulated inflammatory cytokines can significantly inhibit the synthesis of testosterone and affect the synthesis of meiotic DNA in spermatocytes, inducing sperm cell apoptosis [16,17,18].

Testicular interstitial fibrosis is another important cause of testicular damage [19]. Leydig cell fibrosis leads to a decrease in testosterone secretion, reduces sperm quality, and destroys testicular tissue [20]. TGF-β1 is a key fibrotic factor that causes tissue and organ fibrosis. It regulates the transcription of genes by phosphorylating Smad 2/3 protein, which causes testicular tissue fibrosis; therefore, this signaling pathway is vital for inducing the interstitial fibrosis of testes in diabetes [21,22].

Black rice is native to China, and it is known as “black pearl” due to its various dietary and therapeutic functions. Black rice is rich in anthocyanins, and cyanidin-3-glucoside (C3G) accounts for 90% of all anthocyanins in black rice [23]. C3G from black rice has many biological functions, such as anti-oxidation [24], lowering blood lipid [25], anti-inflammatory properties [26], anti-hepatic fibrosis [27], anti-aging properties, etc. [28]. Our previous studies found that black rice C3G has the effects of lowering blood glucose, anti-diabetic nephropathy, and anti-diabetic osteoporosis. It can also inhibit diabetic renal fibrosis via anti-oxidation, anti-inflammation and restraining the TGF-β1/Smad pathway [29,30,31]. Diabetic testicular injury is connected with oxidative stress, apoptosis, inflammation, and fibrosis. We speculated that C3G might have a preventive effect in testicular injury in diabetes.

Here, a type 1 diabetic rat model was established and supplemented with C3G to evaluate its effect on diabetic testicular injury, at the same time revealing its testicular protective mechanism via anti-oxidative stress, anti-inflammation, apoptosis regulation, and the TGF-β1/Smad pathway. This study will provide a theoretical basis for application of C3G in male reproductive damage caused by diabetes.

## 2. Materials and Methods

### 2.1. C3G Preparation

Black rice was provided by Shaanxi Shuangya Grain and Oil Industry and Trade Company (Yangxian, China). C3G was extracted from black rice and characterized using the methods we reported previously [23,29,31]. The purity was >97.4%.

### 2.2. Animals and Diet

Male SD rats (210~223 g, eight weeks old) were provided by Xi’an Yifengda Biotechnology Company. Rats were housed in independent ventilation cages, fed with the AIN93 standard diet in a standard animal housing lab (temperature: 19 °C to 23 °C, air humidity: 36% to 48%). The animal experiment was started after being approved by the Animal Ethics Committee in Shaanxi Daoerfeng Biotechnology Company, Hanzhong, China (No. 20211201).

### 2.3. Type 1 Diabetes Animal Model Induction

The time for adaptive animal feeding was seven days, and then all of the rats were regrouped as control and model groups. Rats in the model were fasted for 12 h and then intraperitoneally injected with streptozotocin (45 mg/kg). Then, 72 h after injection, the blood glucose of each rat was detected; rats with blood glucose levels over 11.1 mmol/L, as well as symptoms of excessive drinking, urination, and overeating, were determined to be diabetic [32].

### 2.4. Animal Grouping and C3G Treatment

**Figure 1:**
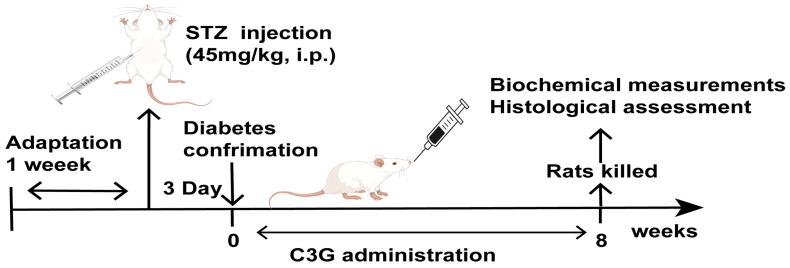
Animal experimental scheme.

The animal experimental process is shown in Figure 1. Rats were regrouped into control, diabetic, C3G-L, C3G-H, and metformin groups after the diabetes rat model was established. Each group contained 12 rats. Rats in the control and diabetic groups were given a vehicle every day. Rats in the C3G-L group and C3G-H group were given C3G (50 or 100 mg/kg/day) via oral gavage. Rats in the metformin group were given metformin 150 mg/kg/day. The blood glucose, body weight, and food and water intake were measured weekly. On the last day of the eighth week, fasting blood glucose was tested from the tail vein. Then, rats were placed under isoflurane anesthesia, cardiac blood was collected, serum was centrifuged (3000 r/min, 10 min) and stored at −80 °C in a refrigerator, testicles were taken out and accurately weighed, and the testis index was calculated as testis weight (mg) divided by rat weight (g). Then, testicular tissues were fixed in paraformaldehyde and some testicular tissues were stored at −80 °C.

### 2.5. Sperm Count, Motility and Viability

Epididymis was taken out and put it into 100 mL preheated DMEM at 37 °C. Then, the epididymis was cut open so that the sperm could be fully released into the culture medium at 37 °C. Computer-assisted semen analysis system (Jiangsu Zhuyue Biotechnology Co., Ltd., Beijing, China) was used to detect sperm concentration, motility rate, and viability.

### 2.6. Sperm Abnormality Assessment

Sperm smears were conducted using fresh semen collected from rats’ epididymis. After slide drying, a drop of methanol was added on the slide to fix the sperm, and then slides were stained using eosin for 25 min, washed with tap water, and dried at room temperature. Stained sperm slides were observed and sperm was counted (total sperm count and abnormal sperm count) under a microscope from 20 visual fields in each slide. The sperm abnormality rate was calculated as the number of abnormal sperm divided by the total sperm.

### 2.7. Biochemical Assay in Serum and Testis Homogenate Samples

A microplate reader (FLX800, Bio Tex, Pearland, TX, USA) and spectrophotometer photometer (YuanXiV-5800, Shanghai, China) were used to detect all indicators. Testosterone, LH, FSH, TNF-α, IL-1β, IL-6, GPX, SOD, MDA, and CAT (Wuhan Saipei Biotech Co., Ltd., Wuhan, China) levels were detected in serum or testis homogenate samples. All operations are based on the ELISA kit instructions.

### 2.8. Histology Evaluation of Testicles

The left testicle of each rat was used to make tissue slices, cleaned after sampling, and fixed with 4% paraformaldehyde for 48 h. Then, testicle tissues were dehydrated by gradient ethanol for one hour and soaked in xylene. Next, the testicular tissue was soaked in melted paraffin (60 °C) for 2 h, and the tissue was fixed in paraffin, cut into 4 μm thick sections, and dried. After that, testicular sections were stained by H&E (hematoxylin and eosin) and observed under a microscope (Leica DM 3000, Leica Microsystems, Wetzlar, Germany). Ten view fields were randomly selected for each slice and scored using Johnsen’s score [33]. Detailed guidelines are shown in Table 1.

### 2.9. Masson Staining for Testicular Interstitial Fibrosis Evaluation

The prepared testicular section was stained with Masson’s Trichrome dye to show the fibrous connective tissues in testis [34]. The ratio between the area of fibrosis tissue and the total tissue area was assessed using Image Pro-Plus 6.0 software under a microscope.

Testicular interstitial fibrosis area percent = (fibrotic area) / (the total area) × 100%(1)

### 2.10. PAS Staining to Observe Testicular Seminiferous Tubules Basement Membrane

Next, 4 μm thick testicular tissue sections were stained using a Periodic Acid-Schiff Staining Kit (Gefan Biotechnology Company, Shanghai, China) according to the kit instructions, and the seminiferous tubule basement membrane changes in testis in each group were observed under a microscope (Leica DM 3000); seminiferous tubule basement membrane thickness was measured using Image Pro-Plus 6.0.

### 2.11. Ultrastructure Observation of Testicular Tissue

Rat testicular tissues were fixed in glutaraldehyde, dehydrated with gradient ethanol, and coated with epoxy resin. Then, the testicular tissues were cut into ultra-thin slices (70 nm thick). Ultra-thin slices of testicular tissues were stained using 0.5% uranium-dioxide acetate and 0.25% citric lead acid. The ultrastructural changes in testis were observed by transmission electron microscopy.

### 2.12. Immunohistochemical Analysis

Then, 4 μm thick testicular slides were dipped in xylene, gradient ethanol, 1% TitinX-100, and 0.3% H_2_O_2_ 3% BAS for 30 min. Next, a primary antibody (TGF-β1, Bcl-2, Bax, p-Smad 2, Caspase-3, p-Smad 3) was added on the testicular slide and incubated in a wet box in incubator for 2 h (37 °C). Next, a secondary antibody was added on the slides, which were stored at 37℃ for 1 h, before adding DAB to the slides. Positive staining areas of the proteins (TGF-β1, Bcl-2, Bax, p-Smad 2, Caspase-3, p-Smad 3) were observed using a microscope and analyzed with Image Pro Plus 6.0 software.

### 2.13. Statistical Analysis

One-way analysis of variance (ANOVA) and Tukey’s test were used to analyze significant differences between any two groups. The above analysis was made using SPSS software (version 22.0).

## 3. Results

### 3.1. C3G Lowered Blood Glucose, Water and Food Intake, Increased Weight of Diabetic Rats

**Figure 2:**
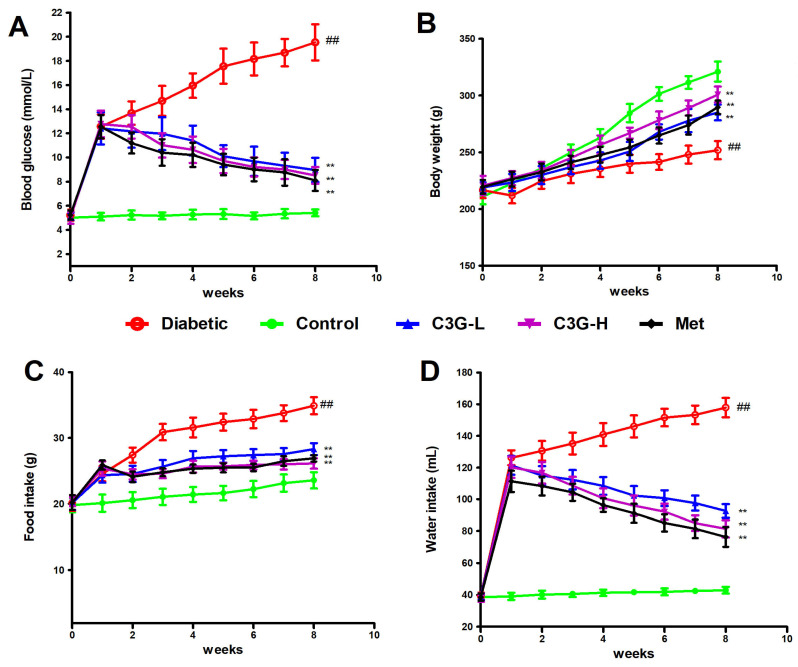
Physiological indexes of diabetic rats. (**A**) Blood glucose. (**B**) Body weight. (**C**) Food intake. (**D**) Water intake. ^##^ *p* < 0.01 vs. control, ** *p* < 0.01 vs. diabetic group.

As Figure 2 shows, blood glucose, water, and food intake significantly increased, and body weight significantly decreased in diabetic animals (vs. control, *p* < 0.01). In C3G or the metformin group, decreased blood glucose, water and food intake levels, and weight gain were observed in diabetic rats. Significant differences exist in those indexes between C3G- or metformin-treated animals and diabetic animals (*p* < 0.01).

### 3.2. C3G Increased the Testicular Index and Repaired the Sperm Quality of Diabetic Rats

**Figure 3:**
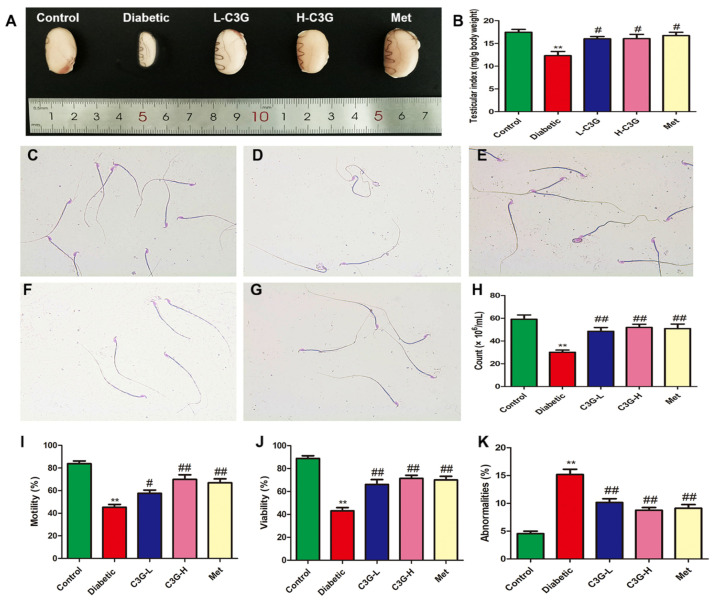
C3G improves testicular index and sperm quality in diabetic rats. (**A**) Gross anatomy of testis. (**B**) Testicular index. (**C**) Control group of sperm smear. (**D**) Diabetic group of sperm smear. (**E**) C3G-L group of sperm smear. (**F**) C3G-H group of sperm smear. (**G**) Met group of sperm smear. (**H**) Sperm count in each group. (**I**) Sperm motility in each group. (**J**) Sperm viability in each group. (**K**) Sperm abnormality rate in each group. ** *p* < 0.01 vs. control, ^#^ *p* < 0.05 vs. diabetic group, ^##^ *p* < 0.01 vs. diabetic group.

In Figure 3, compared with the healthy rats, the testicles of diabetic rats were atrophied; the testicular index decreased; the sperm number, motility, and sperm viability in diabetic group all significantly decreased; and the rate of sperm abnormalities was significantly increased. There were many sperm with big heads, tail curls, and broken tails. After 8 weeks of C3G or metformin administration, sperm number, motility, and viability improved, and the sperm abnormality rate reduced (vs. diabetic group, *p* < 0.01).

### 3.3. C3G Supplementation Relieved Testicular SOD, GPX, MDA, and CAT of Diabetic Rats

**Figure 4:**
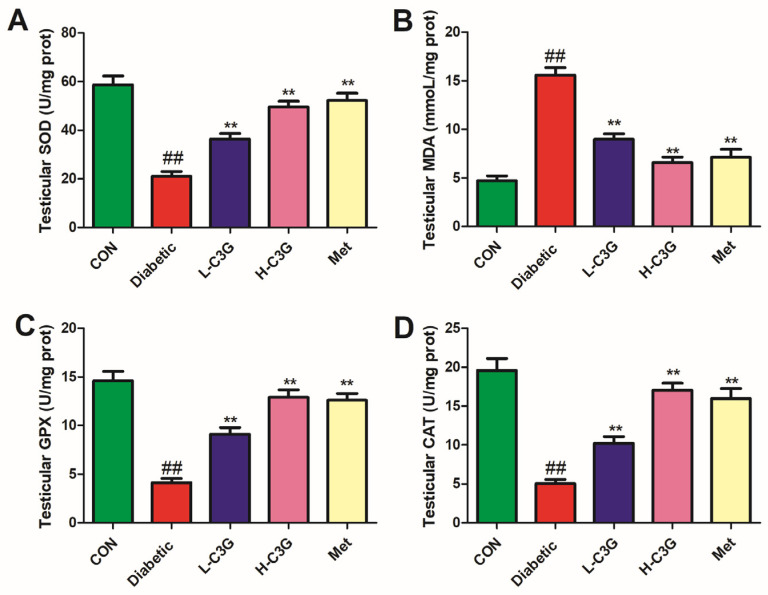
C3G alleviates SOD, GPX, MDA, and CAT indexes in the testicles of diabetic rats. (**A**) Testicular SOD activity. (**B**) Testicular MDA levels. (**C**) Testicular GPX activity. (**D**) Testicular CAT activity. ^##^ *p* < 0.01 vs. control, ** *p* < 0.01 vs. diabetic group.

In Figure 4, the SOD, GPX, and CAT levels in the testis of diabetic rats dramatically reduced, whereas the MDA level was significantly enhanced (vs. control, *p* < 0.01). The testicular tissue of the C3G-treated group and metformin group displayed significantly higher SOD, GPX, and CAT levels (*p* < 0.01, vs. diabetic group), and a lower MDA level (vs. diabetic group, *p* < 0.01).

### 3.4. C3G Up-Regulated the Testosterone, LH, and FSH Content of Serum in Diabetic Rats

Compared with the control group, serum testosterone, LH, and FSH levels were lowered in the diabetic group (*p* < 0.01) (Table 2), and were then increased after eight weeks of C3G or metformin treatment (*p* < 0.01, vs. diabetic group), which showed that C3G could increase testicular hormone levels.

### 3.5. C3G Reduced Inflammatory Cytokines in Serum and Testicular Tissue of Diabetic Rats

**Figure 5:**
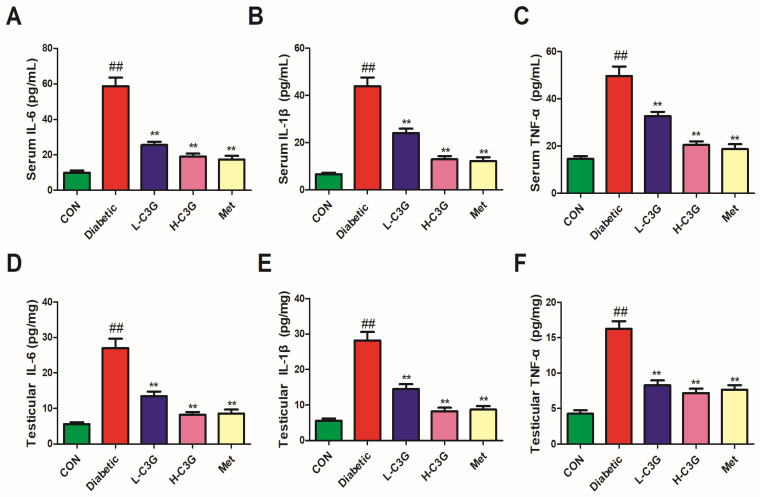
C3G relieves the serum and testicular inflammatory cytokines of diabetic rats. (**A**) Serum IL-6 level. (**B**) Serum IL-1β level. (**C**) Serum TNF-α level. (**D**) Testicular IL-6 level. (**E**) Testicular IL-1β level. (**F**) Testicular TNF-α level. ^##^ *p* < 0.01 vs. control, ** *p* < 0.01 vs. diabetic group.

As indicated in Figure 5, IL-6, IL-1β, and TNF-α levels, both in serum and testicular tissues of diabetic rats, were significantly elevated (*p* < 0.01, vs. control). Those were all lowered after C3G or metformin supplementation, and significant differences were found (vs. diabetic group, *p* < 0.01), which indicates that C3G has an inhibitory effect on testicular tissue inflammation caused by diabetes.

### 3.6. C3G Repaired Pathological Structure of Testicular Tissue of Diabetic Rats

**Figure 6:**
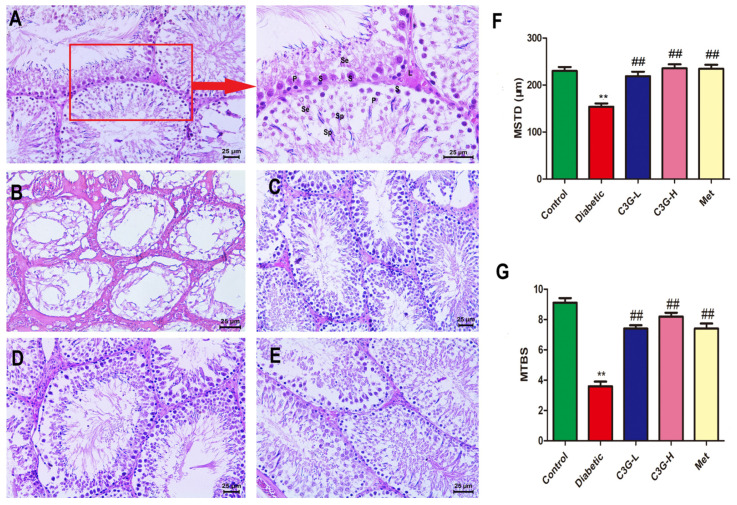
Effect of C3G on structure of testicular tissue of diabetic rats. (**A**) Testicular tissue of control group rat: S. Spermatogonlum, P. Primary spermatocyte, Se. Secondary spermatocyte, Sp. Spermatids, L. Leydig cells. An enlarged picture of the red box is shown in the direction indicated by the red arrow. (**B**) Testicular tissue of diabetic group rat. (**C**) Testicular tissue of C3G-L group rat. (**D**)Testicular tissue of C3G-H group rat. (**E**) Testicular tissue of Met group rat. (**F**) The mean seminiferous tubule diameter (MSTD) in each group. (**G**) Johnsen’s mean testicular biopsy score (MTBS) in each group. ** *p* < 0.01 vs. control, ^##^ *p* < 0.01 vs. diabetic group.

In Figure 6, HE staining revealed that in the control group (Figure 6A), spermatogonia were neatly arranged in seminiferous tubules, and a large number of spermatocytes were clearly visible, as well as sperm cells. In the diabetic group (Figure 6B), the morphology of seminiferous tubules was obviously damaged; there were no sperm cells in the seminiferous tubules, and the mean testicular biopsy score (MTBS) and mean seminiferous tubule diameter (MSTD) were reduced (vs. control, *p* < 0.01). As shown in Figure 6C–E, the morphology of seminiferous tubules were repaired after 8 weeks of C3G or metformin supplementation. As indicated in Figure 6F,G, the MSTD and MTBS were increased by C3G or metformin treatment (C3G-L, C3G-H, and Met group vs. diabetic group, *p* < 0.01), which indicated that C3G could repair testicular pathological damage in diabetic rats.

### 3.7. C3G Inhibited the Testicular Interstitial Fibrosis of Diabetic Rats

**Figure 7:**
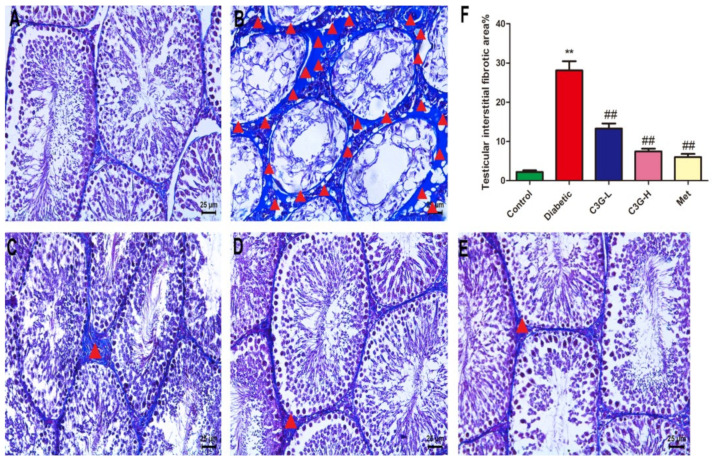
Effect of C3G on testicular interstitial fibrosis of diabetic rats. Testicular tissues were stained with Masson. (**A**) Testicular tissue of control group rat. (**B**) Testicular tissue of diabetic group rat. (**C**) Testicular tissue of C3G-L group rat. (**D**) Testicular tissue of C3G-H group rat. (**E**) Testicular tissue of Met group rat. (**F**) The percentage of testicular interstitial fibrosis area in each group. The red triangle refers to the area of testicular interstitial fibrosis. ** *p* < 0.01 vs. control, ^##^ *p* < 0.01 vs. diabetic group.

In Figure 7, the percentage of testicular interstitial fibrosis area in diabetic group was significantly higher than that in the control (*p* < 0.01). As shown in (Figure 7C–E), after eight weeks of C3G or metformin treatment, the area of fibrosis in testicular interstitium was significantly reduced, and the percentage of testicular interstitial fibrosis area significantly decreased (C3G-L, C3G-H, and Met group vs. diabetic group, *p* < 0.01), which showed that C3G could inhibit testicular interstitial fibrosis of diabetic rats.

### 3.8. C3G-Repaired Morphology of the Seminiferous Tubule Basement Membrane of Diabetic Rats

**Figure 8:**
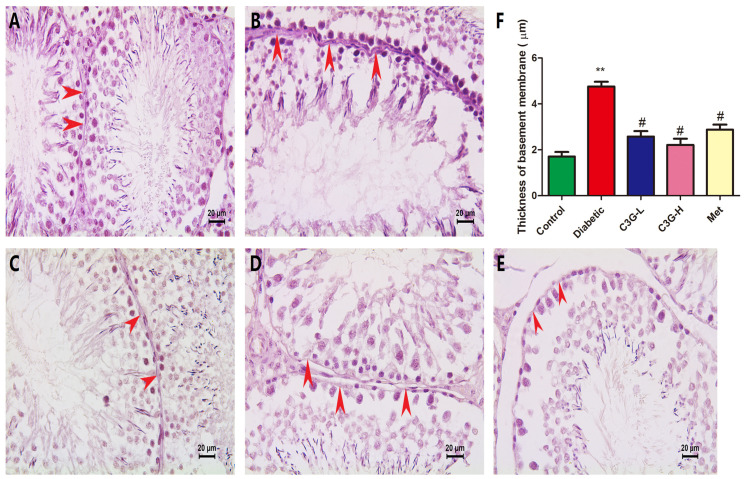
The effect of C3G on the seminiferous tubule basement membrane of diabetic rats. Testicular tissues were stained with PAS. (**A**) Testicular tissue of control group rat. (**B**) Testicular tissue of diabetic group rat. (**C**) Testicular tissue of C3G-L group rat. (**D**) Testicular tissue of C3G-H group rat. (**E**) Testicular tissue of Met group rat. (**F**) The thickness of seminiferous tubule basement membrane in each group. The red arrows point to the seminiferous tubule basement membrane. ** *p* < 0.01 vs. control, ^#^ *p* < 0.05 vs. diabetic group.

The PAS staining of the testis was shown in Figure 8, the seminiferous tubule basement membrane (STBM) in the control group rat was normal (Figure 8A), whereas the thickened STBM was observed in diabetic rats (Figure 8B), and the structure of STBM in C3G or metformin groups became normal (Figure 8C–E). As shown in Figure 8F, STBM thickness significantly increased in the diabetic group (vs. Control, *p* < 0.01) and significantly decreased in the C3G- or metformin-treated groups (vs. diabetic group, *p* < 0.01).

### 3.9. C3G-Repaired Ultrastructure of Testis of Diabetic Rats

**Figure 9:**
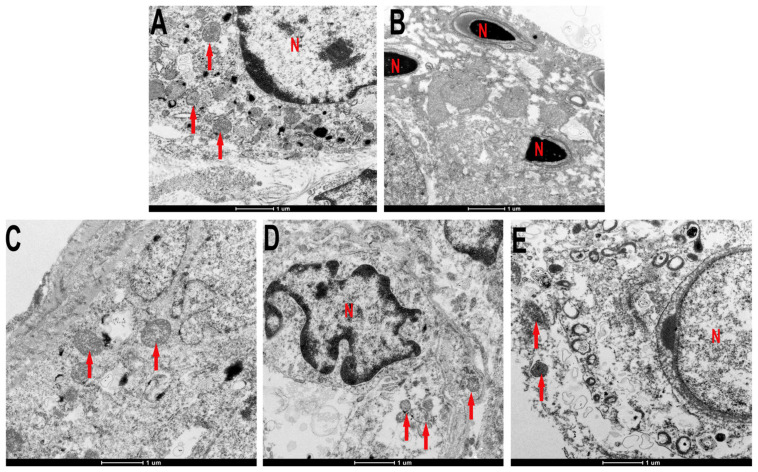
The effect of C3G on the ultrastructure of testis in diabetic rats. Transmission electron microscope (TEM) of testicular tissue. (**A**) Testicular TEM of control group rat. (**B**) Testicular TEM of diabetic group rat. (**C**) Testicular TEM of C3G-L group rat. (**D**) Testicular TEM of C3G-H group rat. (**E**) Testicular TEM of Met group rat. The red arrows point to mitochondrion, N. nucleus.

As Figure 9A shows, the regular ultrastructure of testicles was displayed in the control group, seminiferous tubules were complete, and the nuclear membrane was clear. In addition, the sertoli cell nucleus was large and clear, and the cytoplasm contained a large number of mitochondria. As indicated in Figure 9B, ultrastructural pathological changes existed in the testicles of diabetic rats, organelles in the cytoplasm were dissolved, the nuclear membrane was indistinct, the mitochondrial crest was broken and decreased, the nuclei were pyknotic, and the chromatin edges were concentrated. The ultrastructure of testis in C3G- or metformin-treated groups became normal, as indicated in Figure 9C–E.

### 3.10. C3G Regulated Cell-Apoptosis-Related Proteins in the Testis of Diabetic Rats

**Figure 10:**
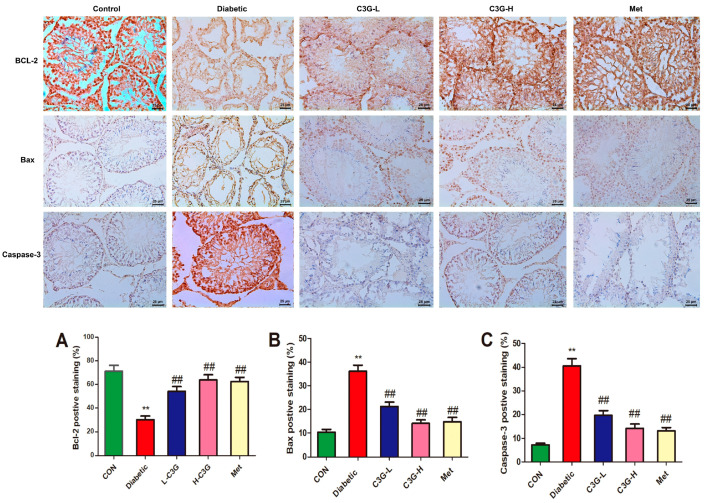
Effect of C3G on Bcl-2, Bax, Caspase-3 protein expression in the testis of diabetic rats. (**A**) Testicular tissue expression of Bcl-2 in each group. (**B**) Testicular tissue expression of Bax of each group. (**C**) Testicular tissue expression of Caspase-3 of each group. ** *p* < 0.01 vs. control, ^##^ *p* < 0.01 vs. diabetic group.

In Figure 10, the levels of Bax and Caspase-3 protein expression in diabetic rat testicles increased (vs. Control, *p* < 0.01); however, these levels were decreased by C3G or metformin supplementation (C3G-L, C3G-H, and Met group vs. diabetic group, *p* < 0.01). In the diabetic group, the Bcl-2 expression level was lowered (vs. Control, *p* < 0.01), and it was up-regulated by C3G or metformin supplementation (vs. diabetic group, *p* < 0.01).

### 3.11. C3G Down-Regulated TGF-β1/Smads Pathway in the Testis of Diabetic Rats

**Figure 11:**
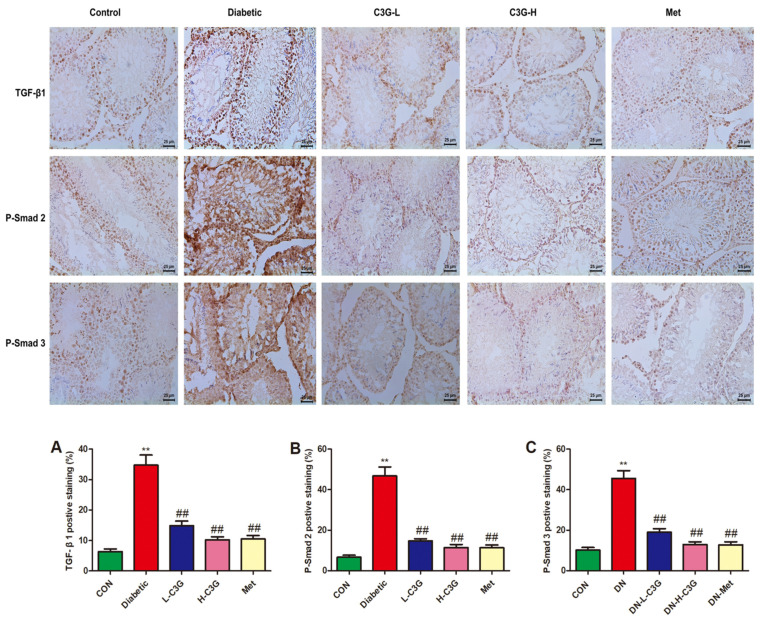
Regulating effects of C3G on TGF-β1, p-Smad2, and p-Smad3 protein expression in the testis of diabetic rats. (**A**) Testicular tissue expression of TGF-β1 in each group. (**B**) Testicular tissue expression of p-Smad2 in each group. (**C**) Testicular tissue expression of p-Smad3 in each group. ** *p* < 0.01 vs. control, ^##^ *p* < 0.01 vs. diabetic group.

As Figure 11 shows, the protein expression levels of TGF-β1, p-Smad2, and p-Smad3 were greatly up-regulated in diabetic group (vs. Control, *p* < 0.01), and they were down-regulated following 8 weeks of C3G-L and C3G-H administration. As indicated in Figure 11A–C, the positive staining percentages of TGF-β1, p-Smad2, and p-Smad3 all significantly decreased in the C3G- or metformin-treatment groups (C3G-L, C3G-H, and Met group vs. diabetic group, *p* < 0.01).

## 4. Discussion

Type 1 diabetes is characterized by a relatively rapid onset, with clear symptoms such as excessive drinking, urination, overeating, fatigue and weight loss. In this study, streptozotocin (with the dosage of 45 mg/kg) was used to induce type 1 diabetes, 72 h after STZ injection, the rats showed an increase in food intake, water consumption and urine output, and a sharp increase in blood glucose, which was consistent with the clinical symptoms of type 1 diabetes, and many other investigators also use a single dose of 45 mg/kg to establish type 1 diabetes [35,36].

Diabetic testicular damage is a very common complication in type 1 and type 2 diabetic men, and about 90% of diabetic men have varying degrees of reproductive dysfunction. The natural fertility rate of type 1 diabetes men is only 50%, especially those who have been ill for a long time [8]. The testicles have two major physiological functions, testosterone secretion and sperm production. Testicular weight, testosterone level, sperm number, sperm motility, sperm viability, as well as testicular morphological structure are all important indicators of reproductive function in male animals [37,38]. In the present study, diabetic-induced testicular damage has many typical symptoms, such as testicular atrophy, decreased sperm number, decreased sperm motility, increased deformity rate, and testosterone disorders, etc. These symptoms all appeared in diabetic rats, indicating that the established diabetes model was successful. Here, after black rice C3G intervention, the pathological damage to testicular tissues, and the number or motility of spermatozoa as well as testosterone levels, were greatly restored in type 1 diabetic rats, these indicated that C3G has a preventive effect on testicular injury in diabetes. The C3G extracted from black rice is the same as the commercially available C3G. Therefore, this research will expand the application of C3G in testicular injury in diabetes.

Many studies have reported that oxidative stress causes testicular damage in diabetes, leading to lowered sperm quality and disturbed sex hormone levels [39,40]. During diabetes, excessive ROS can cause damage to germ cells. Sperm are vulnerable to ROS because their cell membranes have unsaturated fatty acids. Oxidative stress can damage the structure of sperm DNA, and accelerate sperm cell apoptosis, thereby reducing sperm number and mobility, hindering normal sperm development, and impairing their function and inhibition of testosterone secretion, leading to reproductive hormone disorder [41,42,43]. Moreover, in diabetes, ROS produced in sperm can induce lipid peroxidation and destroy sperm nuclei and mitochondrial DNA, thus reducing sperm vitality and quantity and increasing sperm abnormality rates [44,45]. In vivo studies showed that many antioxidants, such as selenium, vitamin C, and taurine, have beneficial effects on diabetic testicular spermatogenesis [46,47]. In the present study, after eight weeks of C3G treatment, sperm count, sperm motility, and sperm viability greatly increased, serum and testicular antioxidant enzyme (SOD, GPX, CAT) activities significantly increased, and MDA level decreased, which indicated that C3G has an anti-oxidative stress effect, which is one of the mechanisms of its testicular protection effect in diabetic rats.

Inflammation caused by diabetes is related to many complications, male infertility being one of them. It is well documented that diabetes is related to oxidative stress and inflammation, which increases pro-inflammatory cytokines levels [48]. Testosterone is an important hormone in male animals that promotes sperm production. When it binds to androgen receptors, it can promote sperm production. Studies have shown that pro-inflammatory cytokines can prevent testosterone production, thus inhibiting spermatogenesis [49,50]. Many studies found that anti-inflammatory substances, such as herbs can improve reproductive function by suppressing inflammation, oxidative stress and increasing testosterone production [51]. In our study, C3G decreased serum and testicular TNF-α, IL-6, and IL-1β levels, indicating that C3G has anti-inflammation effects. Testosterone, LH, and FSH levels were dramatically up-regulated with the decrease in inflammatory cytokines, suggesting that C3G restores testicular function by inhibiting inflammation in diabetic rats.

On the other hand, it was reported that in diabetes, the body produces a large amount of ROS, inducing the formation of NLRP3 inflammatory bodies. NLRP3 prompts IL-1β, IL-18, and IL-6 maturity, and eventually caspase-1 takes its effect, ultimately inducing sperm cell apoptosis, leading to male reproductive dysfunction [52]. In our study, we did not detect ROS and NLRP3, but higher levels of MDA, IL-1β, and IL-6 were found, as well as a sharp increase in caspase-1 levels and sperm cell apoptosis in diabetic rats, which also confirmed that oxidative stress and inflammation induced by diabetes can lead to the apoptosis of testicular cells. All of these factors were relieved by C3G treatment. The mechanism of testicular injury in diabetes remains unclear. Based on the existing literature and our research, it might be that the antioxidant and anti-inflammation effects of C3G can promote the production of testosterone in the testes of diabetes rats, inhibit the apoptosis of testicular cells, and thus promote spermatogenic function. Further in-depth research is needed to reveal its detailed mechanism.

Testicular interstitial cell apoptosis is found to be involved in diabetes-induced testicular injury, and many studies have reported increased interstitial cell apoptosis in diabetic rat models [53,54]. The major function of Leydig cells is to secrete testosterone; the apoptosis of testicular interstitial cells can lead to decreased testosterone secretion, decreased spermatogenic function and infertility [55]. Physiologically, moderate apoptosis can be observed in normal testis, playing an important role in discarding depleted cells, thus maintaining the interstitial cell population as well as testosterone levels. However, excessive interstitial cell apoptosis may reduce testosterone levels, leading to spermatogenic cell apoptosis and infertility [56]. Many studies also found decreased testosterone levels, increased apoptosis rate, up-regulated Bax, Caspase-3 levels, and up-regulated Bcl-2 levels in diabetic rats [42,57], which was consistent with this study. In the present study, immunohistochemical technology was used to evaluate the expression of apoptosis related proteins, in diabetic rats, elevated Bax and Caspase-3 levels, and lowered Bcl-2 level was discovered in testis, which recovered to normal levels after eight weeks of C3G supplementation, revealing that C3G could inhibit testicular cell apoptosis, which was one of the important mechanisms that C3G exerts anti-testicular damage in diabetic rats. Immunohistochemistry has the advantages of more direct and accurate localization and high qualitative sensitivity, whereas Western blot has the advantage of more accurate quantification. We will conduct further animal experiments in the future studies to explore the optimal dosage of C3G, as well as the synergistic effect of metformin and C3G in diabetic testicular damage. We will also use Western blot to verify these apoptosis proteins, and the mitochondria ability of sperm also needs to be checked.

It was reported that hyperglycemia can cause testicular interstitial fibrosis in diabetic men [58]. In the present study, testicular fibrosis was observed in diabetic rats. Testicular fibrosis is a common pathological change in diabetes, which induced azoospermia and severe oligospermia, and it is a key to damage to the microenvironment of testicular spermatogenesis [59]. TGF-β1 was a vital factor inducing tissue fibrosis and promoting the phosphorylation of Smad proteins [19]. The dysregulation of the TGF-β1/Smad pathway was confirmed as a vital cause of tissue fibrosis and diabetes-induced testicular fibrosis [60]. Smad2 and Smad3 were two important regulators that promoted TGF-β1-mediated tissue fibrosis [61]. In the present study, C3G inhibited testicular TGF-β1, Smad2, and Smad3 expression in diabetic rats, indicating that C3G played an anti-testicular interstitial fibrosis role by regulating the TGFβ1/Smad pathway. This is one of the reasons why C3G improved the microenvironment of testicular spermatogenesis in diabetic rats, as well as being one of the most important mechanisms by which C3G exerts anti-testicular damage in diabetic rats.

## 5. Conclusions

In summary, this research revealed that C3G could increase testicular index, restore pathological changes in testicles, improve sperm quality, inhibit testicular fibrosis, and attenuate STZ-induced type 1 diabetic rat testicular tissue injury. The possible mechanism for this might be that C3G improved antioxidant capacity, relieved oxidative stress, increased anti-inflammatory cytokine expression, and inhibited the apoptosis of spermatogenic cells and testicular fibrosis, thus promoting the production of testosterone and the repair of testicular function. This study provides a theoretical basis for the application of C3G in male reproductive damage caused by diabetes. The mechanism of testicular injury in diabetes is complex and remains unclear, and further in-depth research is needed on its detailed mechanisms of action.

## Figures and Tables

**Table 1:** Mean testicular biopsy score (MTBS) classification.

Score	Description
1	No cells
2	Sertoli cells without germ cells
3	Only spermatogonia
4	Only a few spermatocytes
5	Many spermatocytes
6	Only a few early spermatids
7	Many early spermatids without differentiation
8	Few late spermatids
9	Many late spermatids
10	Full spermatogenesis

**Table 2:** Levels of serum LH, FSH, and testosterone in each group.

Groups	LH (IU/L)	FSH (IU/L)	Testosterone (ng/mL)
Control	1.36 ± 0.21	16.75 ± 1.46	1.29 ± 0.13
Diabetic	0.74 ± 0.14 **	8.36 ± 1.21 **	0.36 ± 0.09 **
L-C3G	1.15 ± 0.18 ^#^	13.28 ± 1.31 ^##^	0.96 ± 0.14 ^##^
H-C3G	1.25 ± 0.22 ^##^	14.22 ± 1.90 ^##^	1.26 ± 0.10 ^##^
Met	1.36 ± 0.19 ^##^	13.44 ± 1.57 ^##^	1.22 ± 0.13 ^##^

** *p* < 0.01 vs. control group, ^#^ *p* < 0.05 vs. diabetic group, ^##^ *p* < 0.01 vs. diabetic group.

## Data Availability

Data are contained within the article.
